# A review of guard cell wall architecture in shaping stomatal morphology and function

**DOI:** 10.3389/fpls.2026.1815192

**Published:** 2026-04-15

**Authors:** Chuanlei Xiao, Huimin Guo

**Affiliations:** State Key Laboratory Incubation Base for Conservation and Utilization of Bio-Resource in Tarim Basin, College of Life Science, Tarim University, Alar, Xinjiang, China

**Keywords:** drought tolerance, guard cell wall, Stomatal dynamics, Stomatal morphology, water-use efficiency

## Abstract

Stomata serve as essential gateways for gas exchange and water loss, accounting for over 90% water loss through transpiration. Therefore, modulating stomatal movement and development is a key strategy for improving crop water-use efficiency (WUE) and drought tolerance. Emerging evidence highlights the critical role of guard cell wall (GCW) composition and modifications in shaping stomatal morphology and modulating stomatal responses to environmental cues, thereby significantly affecting plant WUE, drought resistance, and photosynthetic performance. This review systematically summarizes recent progress in the regulation of GCW components and their modifications. Furthermore, we discuss the potential involvement of transcriptional cascades and cell wall integrity (CWI) sensing pathways in controlling stomatal morphology and dynamics. These insights offer a theoretical basis for the targeted genetic manipulation of GCWs to concurrently enhance crop WUE and drought resilience.

## 1 Introduction

Stomatal pores are critical for mediating gas exchange between leaf internal air spaces and the atmosphere, substantially influencing plant light- and water-use efficiencies (Wang et al., 2022; Nguyen et al., 2023). Extensive research has targeted genes governing stomatal development (e.g., *EPF*s) and movement (e.g., *GORK*, *BLINK1*) to enhance these efficiencies under diverse environmental conditions. Increasing evidence indicates that plant cell walls (CWs) function as active regulators of stomatal dynamics, development, and photosynthetic performance, rather than serving as passive structural elements.

Recent conceptual frameworks suggest that CW composition directly limits stomatal conductance (*g*_s_) and internal CO_2_ diffusion, thereby modulating photosynthesis and WUE independently of stomatal density, for instance, alterations in CW hydroxycinnamic acid content via overexpression of the CoA acyltransferase gene *OsAT10* affect *g*_s_, mesophyll conductance, and canopy-level WUE (Pathare et al., 2023). Additionally, CW mechanics mediate dynamic stress responses; mutation of the pectin-modifying polygalacturonase gene *PSL1* (*PHOTO-SENSITIVE LEAF ROLLING 1*) alters leaf mechanical properties, facilitating light- and drought-induced leaf rolling that reduces water loss at the expense of growth (Zhang et al., 2021). Furthermore, CW biosynthesis and remodeling are integral to stomatal patterning; disruption of the CW-associated enzyme ZmIRX15A (IRREGULAR XYLEM 15A) results in altered stomatal density and morphology, impacting transpiration and WUE (Zhang et al., 2023a). Given the central role of the CW in stomatal regulation, the GCW warrants particular attention for its specialized structural features that enable precise stomatal movements. These include uneven thickening (dorsal wall thicker than ventral) that directs bulging upon water uptake, radially arranged cellulose microfibrils that confer anisotropic deformation, pectin-rich matrices that provide reversible elasticity, and an outer cuticle that minimizes water loss. Collectively, these adaptations underpin efficient stomatal regulation (Rui et al., 2018).

Taken together, these studies propose the plant CW as a central coordinator of stomatal architecture, dynamics, and photosynthetic performance, integrating developmental and biophysical mechanisms to optimize gas exchange between plants and their environments. Targeted optimization of plant CWs, particularly those of guard cells (GCs), to regulate stomatal behavior while accounting for *g*_s_, mesophyll conductance, and leaf hydraulic conductance, constitutes a promising but underexplored avenue for improving crop WUE.

## 2 Guard cell wall properties modulate stomatal dynamics

Recent biomechanical and computational investigations have reconceptualized GCWs from passive enclosures to dynamic flexoskeletons that govern stomatal movement via anisotropic deformation (Yi et al., 2019). A key determinant of this behavior is the orientation of cellulose microfibrils. It is well established that high-tensile circumferential cellulose fibers convert turgor pressure into the kidney-shaped deformation essential for stomatal opening in eudicots such as Arabidopsis (Woolfenden et al., 2017). Computational models reveal that perturbations to cellulose networks or the hemicellulose (xyloglucan) tethers that bind them, profoundly alter GCW stiffness and stomatal aperture, despite the nonlinear correlation between GCW stiffening and pore area (Yi et al., 2018). Nonetheless, theoretical frameworks underpinning these interpretations remain under critical scrutiny. While some models successfully apply linear elasticity to approximate GC behavior, others contend that this simplification inadequately represents the large deformation and strain-stiffening behavior of the GCW matrix (Woolfenden et al., 2017; Yi et al., 2022). Furthermore, inverse modeling approaches used to infer GCW properties from morphological changes face criticism for potentially yielding non-unique solutions, underscoring the necessity for robust empirical validation (Tan et al., 2025).

A significant conceptual evolution concerns the spatial heterogeneity of GCW stiffness. Whereas traditional models emphasized radial stiffening as imperative for stomatal opening, recent evidence supports a “polar stiffening” paradigm. In Arabidopsis, Carter et al. (2017) demonstrated that stomatal poles exhibit markedly greater stiffness than central regions, proposing that this “fix and flex” architecture, anchored by pectin-rich poles, enhances stomatal opening efficiency. Molecular data from the eudicot *Populus* corroborate this model, implicating the transcription factor MYB156 in modulating polar stiffness via regulation of *PME6* (*Pectin Methylesterase 6*) expression; specifically, targeted pectin de-methylesterification at stomatal poles appears critical for optimal stomatal kinetics in this species (Zheng et al., 2023). However, the functional role of polar stiffening may not be conserved across angiosperms. In contrast to the facilitative effect observed in these eudicots, maize (*Zea mays*) stomata exhibit polar enrichment of methylesterified pectin, cellulose and xyloglucan that restricts stomatal opening, with decreased polar stiffness correlating with increased aperture size (Zhang et al., 2026). This divergence underscores the complexity of GCW architecture across different plant groups and is further supported by the presence of unique symplastic connections in grass stomata, that buffer turgor fluctuations—a feature absent in model eudicots (Jaafar and Anderson, 2024; Wilson et al., 2025).

Taken together, these findings indicate that stomatal dynamics emerge from the complex interplay of GCW polymer biosynthesis, enzymatic modification, and geometric constraints, rather than from a single mechanical determinant. Notably, the role of pectin in regulating GCW matrix stiffness remains speculative; meanwhile, mechanical properties inferred from experiments are highly model-dependent and still require independent validation (Tan et al., 2025). Moreover, the extent to which specific regulatory mechanisms are conserved across divergent plant lineages remains an open question that merits further investigation.

## 3 Stomatal dynamics and morphology are regulated by alterations or modifications in GCW composition

Stomatal aperture dynamics and morphology are fundamentally governed by the mechanical properties of the GCW, which are modulated through the biosynthesis, enzymatic modification, and degradation of its polysaccharide constituents (**Table 1**). Baseline pectin biosynthesis establishes the foundation for GCW mechanical properties. Reduced pectin biosynthesis via At*GAUT10/11* mutations paradoxically enhances stomatal responsiveness and closure speed, thereby increasing drought tolerance, while also affecting GC shape (Guo et al., 2021). Lignin deposition, mediated by laccase PtrLAC17, serves as an induced rigidification mechanism that restricts aperture size to enhance drought tolerance (Shen et al., 2025). Calcium-crosslinked homogalacturonan (HG) imparts rigidity; thus, enzymes modifying or degrading HG are essential for stomatal motility. The methylesterification status of HG is a key determinant of GCW elasticity, though its mechanical effects are multifaceted. In Arabidopsis, Amsbury et al. (2016) proposed that pectin demethylesterification is essential for normal stomatal function; *pme6* mutants, which retain highly methyl-esterified pectin, display reduced GCW flexibility and restricted stomatal dynamics. Conversely, Zhang et al. (2023b) demonstrated that excessive demethylesterification of pectin, as caused by PMEI18 (PECTIN METHYLESTERASE INHIBITOR 18) disruption, leads to accumulation of calcium-pectate crosslinks that rigidify the GCW and inhibit stomatal dynamics in Arabidopsis. Thus, Arabidopsis stomatal functionality depends on a balanced pectin esterification state, maintained by antagonistic PME and PMEI activities (Wu et al., 2017). Moreover, Arabidopsis GC-specific CW modifying enzymes such as PME34, PME53, and PME12 exhibit transcriptional responsiveness to abscisic acid (ABA) and heat stress, dynamically adjusting GCW flexibility to optimize transpiration under environmental stress (Huang et al., 2017; Wu et al., 2017, Wu et al., 2022, Wu et al., 2025). Neutral sugar side-chains, particularly arabinans, further modulate GCW plasticity (**Table 1**). In *Commelina communis*, arabinans prevent the formation of rigid HG associations, thereby maintaining GCW flexibility and facilitating stomatal movement (Jones et al., 2003). In Arabidopsis, the degree of arabinan polymerization positively correlates with GCW elasticity; overexpression of *ARAD1* (*ARABINAN DEFICIENT 1*) increases long-chain arabinans, augmenting stomatal opening and photosynthetic carbon assimilation (Carroll et al., 2022). Controlled pectin degradation is also essential for stomatal motility. Pectate lyase PLL12 activity prevents excessive calcium crosslinking in GCWs of Arabidopsis, with *pll12* mutants exhibiting stiffer GCWs and impaired stomatal dynamics (Chen et al., 2021; Hõrak, 2021). Similarly, Arabidopsis polygalacturonase PGX3 facilitates HG depolymerization, ensuring smooth and continuous stomatal closure; *pgx3* mutants display stepwise closure deficits (Rui et al., 2017). Beyond modulating stomatal dynamics in mature GCs, the precise deposition, demethylesterification, and degradation of pectin during stomatal development are critical determinants of proper stomatal formation and expansion, thereby exerting essential control over stomatal morphology (Rui et al., 2019).

**Table 1:** Genes implicated in stomatal dynamics and morphology through regulation of the CW.

Species name	Gene name & publication	Gene function	Biological function
*Arabidopsis thaliana*	*PME6* Amsbury et al. (2016)	Encodes a pectin methylesterase	Required for demethylesterification of the GCW; establishes the mechanical dynamic range necessary for stomatal function.
*Arabidopsis thaliana*	*PMEI18* Zhang et al. (2023b)	Encodes a pectin methylesterase inhibitor	Inhibits PME activity (specifically PME31) to maintain CW pectin esterification; regulates stomatal dynamics and dimension.
*Arabidopsis thaliana*	*PME12* Wu et al. (2025)	Encodes a type-I pectin methylesterase	Catalyzes pectin demethylesterification; regulates stomatal density, pore aperture, and inhibits thermotolerance.
*Arabidopsis thaliana*	*PME53* Wu et al. (2022)	Encodes a type-II pectin methylesterase	Guard cell-specific enzyme required for ABA-mediated stomatal closure and regulating heat stress response.
*Arabidopsis thaliana*	*PME34* Huang et al. (2017)	Encodes a pectin methylesterase	Modulates GCW flexibility; essential for stomatal movement and heat tolerance.
*Arabidopsis thaliana*	*ARAD1* (*ARABINAN DEFICIENT 1*) Carroll et al. (2022)	Arabinan synthase	Synthesizes CW arabinans; increasing arabinan chain length enhances GCW flexibility and allows for wider stomatal opening.
*Arabidopsis thaliana*	*SFR8* (allelic to *MUR1*) Panter et al. (2023)	GDP-D-mannose 4,6-dehydratase	Catalyzes the first step in the *de novo* synthesis of GDP-L-fucose; mutation lowers GCW stiffness, affecting cuticular ledge formation and water retention.
*Arabidopsis thaliana*	*MUR1*, *GFT1* Waszczak et al. (2024)	GDP-D-mannose 4,6-dehydratase; GDP-fucose transporter	*MUR1* synthesizes fucose, *GFT1* transports it to the Golgi; deficiency leads to structural defects (e.g., obstructed pores) and high-water loss independent of stomatal movements.
*Zea mays*	*BZU3* Zhou et al. (2023)	UDP-glucose 4-epimerase	Regulates the supply of UDP-glucose during GCW synthesis; essential for local wall thickening and the formation of the dumbbell-shaped guard cells.
*Arabidopsis thaliana*	*GAUT10*, *GAUT11* Guo et al. (2021)	Encode galacturonosyltransferases	Function in homogalacturonan (pectin) biosynthesis; essential for stomatal development and dynamics (mutants show faster closure and drought tolerance).
*Arabidopsis thaliana*	*CESA3* Rui and Anderson (2016)	Encodes a cellulose synthase isomer	Cellulose biosynthesis.
*Arabidopsis thaliana*	*XXT1*, *XXT2* Rui and Anderson (2016)	Encodes a protein with xylosyltransferase activity	Xyloglucan biosynthesis.
*Arabidopsis thaliana*	*COBL7*, *COBL8* Ge et al. (2024)	COBRA-like membrane-anchored cell wall proteins	Required for stomatal formation via regulation of cellulose deposition.
*Oryza sativa*	*OsBC1L1*, *OsBC1L8* Li et al. (2021)	COBRA-like GPI-anchored proteins	Regulate stomatal production, patterning, and subsidiary cell formation.
*Populus trichocarpa*	*PtrLAC17* Shen et al. (2025)	Laccase	Catalyzes the polymerization of lignin monomers in GCWs; increased lignin deposition of overexpression lines reduces stomatal aperture size and improves drought tolerance.
*Arabidopsis thaliana*	*PLL12* Chen et al. (2021)	Pectate Lyase	Cleaves pectic homogalacturonan (HG); prevents excessive calcium-mediated cross-linking to maintain wall mechanics required for proper stomatal dynamic range.
*Arabidopsis thaliana*	*PGX3* Rui et al. (2017)	Polygalacturonase	Hydrolyzes HG backbones; modulates pectin molecular mass to facilitate stomatal pore separation in cotyledons and smooth closure dynamics in mature leaves.
*Arabidopsis thaliana*	*SCAP1* Negi et al. (2013)	Encodes a Dof-type transcription factor	Regulates the expression of downstream genes (including *PME*s, *GORK*, and *MYB60*) essential for GC maturation and functional stomatal movement.
*Arabidopsis thaliana*	*MYS1*, *MYS2* Peng et al. (2024)	Encode MYB-like transcription factors	Regulate hoop rigidity and stomatal pore formation by controlling the expression of CW-modifying genes during maturation.

Cellulose and hemicellulose form the load-bearing framework of the GCW and confer anisotropic mechanical properties essential for stomatal movement (**Table 1**). In Arabidopsis, cellulose microfibrils undergo dynamic reorganization during stomatal movements, shifting from a uniform distribution in the open state to a fibrillar pattern in the closed state (Rui and Anderson, 2016). Disruption of cellulose or hemicellulose synthesis impairs this anisotropic property; both the cellulose synthase mutant (*cesa3^je5^*) and the xyloglucan-deficient mutant (*xxt1 xxt2*) exhibit aberrant stomatal apertures, changes in GC length, and defects in cellulose reorganization during stomatal movement. These findings indicate that sufficient cellulose is essential for normal GC dynamics and highlight the importance of xyloglucan-cellulose interactions in GCW function (Rui and Anderson, 2016). Beyond dynamic movement, cellulose organization is critical for establishing functional stomatal morphology (**Table 1**). In Arabidopsis, the glycosylphosphatidylinositol (GPI)-anchored proteins COBL7 and COBL8 regulate cellulose deposition and ventral wall modification; their disruption reduces cellulose content and impairs stomatal pore formation (Ge et al., 2024). In grasses, which possess dumbbell-shaped GCs flanked by subsidiary cells, the GPI-anchored proteins OsBC1L1 and OsBC1L8 play redundant roles in stomatal development; the *osbc1l1 osbc1l8* double mutant exhibits excessive stomatal production, clustering, and defective subsidiary cell formation, demonstrating that proper CW remodeling is critical for stomatal morphology (Li et al., 2021). Additionally, substrate availability for cellulose and hemicellulose biosynthesis is also critical for proper GC morphology; deficiencies in these substrates cause structural collapse, particularly affecting the dumbbell-shaped GCs of grasses without directly altering stomatal kinetics (Zhou et al., 2023). Although cellulose and hemicellulose form the load-bearing framework of GCW and underpin anisotropic mechanics for stomatal movement, the determinants of cellulose microfibril-driven anisotropy remain unclear (Tan et al., 2025). In contrast, certain CW-related modifications primarily influence stomatal morphology and water tightness through effects on structures beyond the GCW itself (**Table 1**). Mutations impairing fucosylation (e.g., in *MUR1/SFR8* and *GFT1*) disrupt the formation of the cuticular ledge, a specialized extracellular structure at the stomatal pore, as well as proper pore sizing. These defects compromise the integrity of the outer cuticular barrier that extends over the GCs and surrounding epidermal cells, resulting in unregulated water loss that occurs independently of abscisic acid (ABA) signaling (Panter et al., 2023; Waszczak et al., 2024). Thus, while GCW composition directly influences stomatal mechanics, water loss phenotypes can also arise from alterations in adjacent CW domains or extracellular structures that are not intrinsic to the GCW itself.

The specialized mechanical properties of the GCW, whether conferred by pectin, cellulose, or lignin, are ultimately orchestrated by transcriptional regulators (**Table 1**). In Arabidopsis, transcription factors such as AtSCAP1 (STOMATAL CARPENTER 1) and AtMYS1/2 (MYB-SHAQKYF 1/2) orchestrate GCW maturation, coordinating establishment of hoop rigidity and pore morphology necessary for functional stomata (Negi et al., 2013; Peng et al., 2024). How these regulators integrate with cell wall integrity (CWI) sensing pathways to modulate GCW dynamics represents an important avenue for future investigation.

Collectively, these findings delineate dual roles for GCW components in GC function: cellulose and fucosylated polymers establish static stomatal morphology, while pectin, arabinan, lignin, and their enzymatic modifications actively modulate mechanical properties to enable or restrict stomatal dynamics.

## 4 Receptor-like protein kinases monitor disruptions or modifications in cell wall components

Recent advances in plant CWI sensing elucidate a complex receptor-like protein kinase (RLK)-mediated network that monitors both biochemical composition and mechanical status of the CW to regulate growth, morphogenesis, biotic and abiotic adaption. A key regulatory mechanism involves the detection of pectin methylesterification, which serves as a molecular switch between developmental and stress-responsive pathways. Wall-Associated Kinases (WAKs) and WAK-like proteins have been identified as direct pectin sensors, exhibiting ligand specificity that varies with physiological context. For example, *Arabidopsis* RFO1 (RESISTANCE TO FUSARIUM OXYSPORUM 1) preferentially binds demethylesterified pectin to coordinate root development and pathogen resistance (Huerta et al., 2023), whereas rice OsWAK11 favors methyl-esterified pectin and functions to suppress brassinosteroid (BR) signaling, thereby restricting cell elongation during photomorphogenesis (Yue et al., 2022).

Beyond chemical cues, the *Catharanthus roseus* RLK1-like kinase FERONIA (FER) functions as a critical mechanosensor linking the extracellular matrix to intracellular signaling. FER directly interacts with demethylesterified pectin via its extracellular malectin domains, thereby transducing mechanical signals (Feng et al., 2018; Duan et al., 2020; Lin et al., 2022; Tang et al., 2022). This interaction is vital for eliciting diverse downstream responses, including activation of ROP6 GTPase signaling that reorganizes cortical microtubules (CMT) during pavement cell (PC) morphogenesis under mechanical stress (Lin et al., 2022; Tang et al., 2022) and cell-specific calcium transients essential for salt stress tolerance in root (Feng et al., 2018). Furthermore, recent findings support a hierarchical organization within CWI signaling pathways governing CW remodeling. In lignin-deficient *ccr-1* mutants, FER mediates upstream CW remodeling and pectic polysaccharide release, whereas WAKs subsequently perceive these released oligogalacturonides—such as tri-galacturonic acid—to induce defense-related gene expression (Liu et al., 2023). This stratified sensing framework enables precise coordination of physiological processes, ranging from polyspermy prevention via FER-dependent nitric oxide (NO) accumulation to immune activation in response to CW damage (Duan et al., 2020; Liu et al., 2023).

It should be emphasized that most of the evidence regarding the roles of FER and WAKs in CWI perception is derived from studies on roots, pavement cells, fertilization processes, or general stress responses. In contrast, direct experimental evidence supporting their involvement in GCs remains scarce. Consequently, caution is warranted when extrapolating this generalized CWI sensing paradigm to GCs. Specifically, the molecular mechanisms through which GCs specifically detect and transduce CWI signals to modulate stomatal dynamics remain to be fully elucidated. Notably, the receptor-like protein kinase MUSTACHES (MUS) localizes proximal to the plasma membrane during stomatal development, where it regulates GCW assembly and cytoskeletal polarity establishment (Keerthisinghe et al., 2015). Moreover, a recent study has shown that osmotic stress in GCs induces the clustering and activation of FER at the plasma membrane, leading to the formation of nanodomains in a manner dependent on its CW-anchored malectin A (malA) domain (Qin et al., 2026). Similarly, under drought conditions, pectin-derived oligogalacturonides (OGs) activate TaWAK5 (a WAK from *Triticum aestivum L.*), which phosphorylates TaSLAC1 (Slow anion channel-associated 1) to promote stomatal closure and enhance drought resistance (Wang et al., 2026). These findings suggest that analogous regulatory mechanisms may underpin CWI monitoring in GCs.

## 5 Discussion

This review integrates a comprehensive, multilayered understanding of stomatal function, emphasizing the GCW as a dynamic flexoskeleton. Current research delineates a defined molecular architecture whereby load-bearing cellulose microfibrils confer anisotropic mechanical properties, while the pectin matrix imparts regulated flexibility to stomatal movements. This architecture is dynamically remodeled by CW-modifying enzymes, including pectin methylesterases and polygalacturonases, whose activities are likely governed by complex transcriptional networks (Negi et al., 2013; Zheng et al., 2023; Peng et al., 2024). Additionally, CW modification and integrity alteration sensing mechanisms have been evaluated. Under biotic and abiotic stress conditions, cell surface receptor-like protein kinases (RLKs) such as FER and WAKs actively perceive changes in the chemical and mechanical properties of the CW. These RLKs transduce extracellular cues into intracellular signaling cascades that modulate cytoskeletal organization, gene expression, and stress responses.

**Figure 1:**
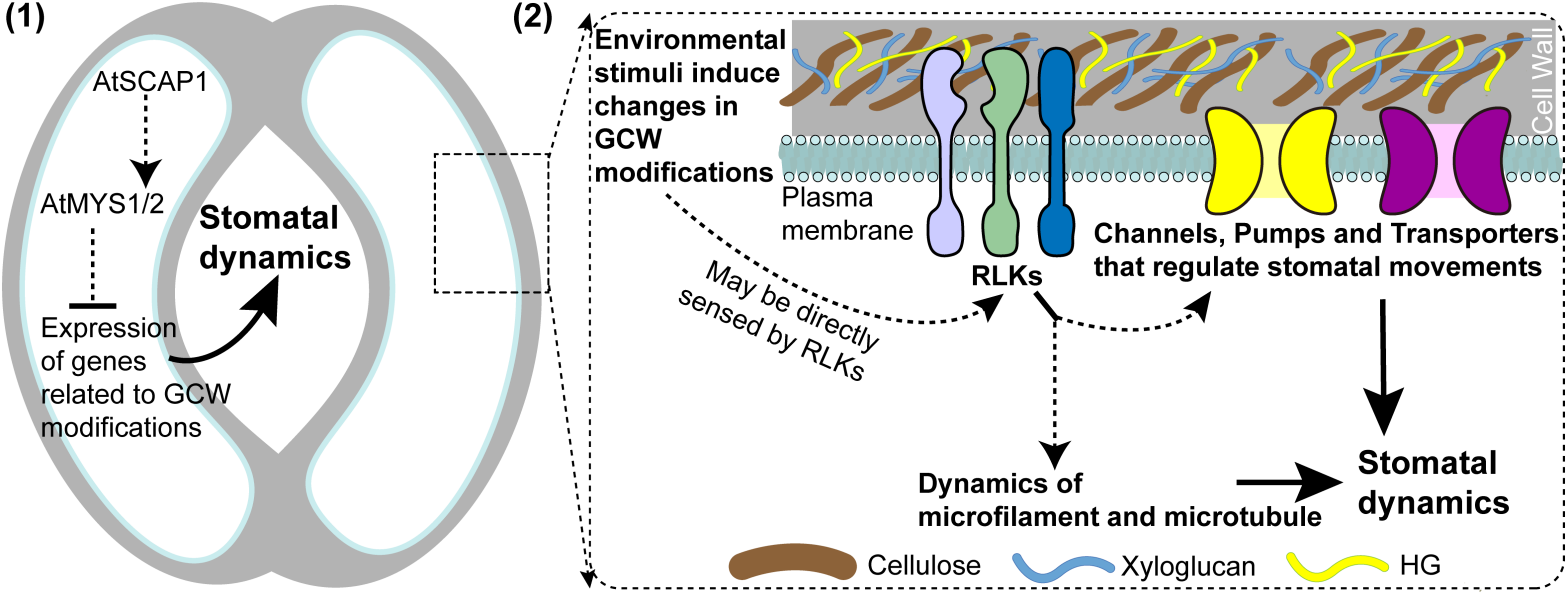
Two hypotheses address guard cell wall (GCW) regulation of stomatal function: (1) the AtSCAP1–AtMYS1/2 transcriptional cascade modulates GCW dynamics to facilitate stomatal movement; (2) receptor-like protein kinases (RLKs) detect GCW modifications and integrity alterations induced by external stimuli, thereby coordinating stomatal responses. Dashed lines indicate potential molecular pathways that require further investigation for validation.

Drawing from the accumulated evidence, two testable hypotheses are proposed regarding GCW-mediated regulation of stomatal dynamics (**Figure 1**). First, transcription factors coordinate stomatal behavior by regulating genes encoding CW-modifying enzymes (**Figure 1**). This is exemplified by MYB156, which represses expression of *PME6*, thereby modulating demethylesterified pectin levels at GC poles to establish the polar stiffening necessary for proper stomatal opening in *Populus* (Zheng et al., 2023). Similarly, transcription factors AtSCAP1, AtMYS1 and AtMYS2 are implicated in establishing hoop rigidity and pore morphology, potentially operating within a shared regulatory pathway to orchestrate the expression of downstream CW modification and degradation genes, thus shaping GCW mechanical properties (**Figure 1**). Second, modifications in GCW composition and CWI of GCs are sensed by RLKs that activate downstream signaling pathways such as cytoskeletal remodeling and ion channel regulation in GCs (Khanna et al., 2014) to control stomatal dynamics (**Figure 1**). This hypothesis is inferred from the known activities of FER and WAKs, which directly bind demethylesterified or methylesterified HG to mediate developmental and defense responses. The receptor-like protein kinase MUS is involved in regulating GCW assembly and cytoskeletal polarity during stomatal development (Keerthisinghe et al., 2015). Meanwhile, FER can be activated by sensing the status changes of the GCW under osmotic stress through its malA domain (Qin et al., 2026); and TaWAK5 can sense OGs derived from GCW pectin degradation to activate TaSLAC1 via phosphorylation under drought stress (Wang et al., 2026). These findings further support this regulatory mechanism, and in-depth research is still required to verify its universality in GCs.
